# ﻿Two new species of the genus *Chilobrachys* (Araneae, Theraphosidae) from China

**DOI:** 10.3897/zookeys.1081.77072

**Published:** 2022-01-13

**Authors:** Yejie Lin, Xunyou Yan, Shuqiang Li

**Affiliations:** 1 Hebei Key Laboratory of Animal Diversity, College of Life Science, Langfang Normal University, Langfang 065000, China Langfang Normal University Langfang China; 2 Institute of Zoology, Chinese Academy of Sciences, Beijing 100101, China Institute of Zoology, Chinese Academy of Sciences Beijing China

**Keywords:** Asia, diagnosis, taxonomy, type

## Abstract

Two new species of the genus *Chilobrachys* Karsh, 1892 are described from China: *Chilobrachysdominus* Lin & Li, **sp. nov.** from Yunnan and *C.jinchengi* Lin & Li, **sp. nov.** from Tibet. Photos and a morphological description of the new species are given. The type specimens of the new species are deposited in the Institute of Zoology, Chinese Academy (IZCAS) in Beijing.

## ﻿Introduction

The family Theraphosidae Thorell, 1869 is one of the largest spider families in the world, including 1031 species in 153 genera (WSC 2021). This family is divided into 12 subfamilies (Foley et al. 2019), of which SelenocosmiinaeSimon 1889 and OrnithoctoninaePocock 1895 are known from Asia.

Selenocosmiinae includes 125 species and subspecies in 12 genera. It can be distinguished from other subfamilies by the maxilla with an oval stridulatory organ on the prolateral surface, originating medially (not ventrally from the oral fringe), the maxilla with opposing modified setae in multiple rows on the retrolateral cheliceral surface, the posterior sternal sigillae distant from the sternal margins, the more than 60 labial cuspules, and the leg spines only found distally on the metatarsi; mature males lack a tibial apophysis on leg I (Nunn et al. 2016). In the genus *Chilobrachys* Karsh, 1892, the anterior eyes are nearly in a straight line, the stridulating organ consists of short spines on the chelicerae and a single or double row of paddle hairs overlapped by a fringe of hairs on the maxillae, and the palpal organ of the male ending in a long and slender embolus (Raven 1985; Zhu and Zhang 2008).

Four *Chilobrachys* species were hitherto known from China (Fig. 8, Li 2020; Li et al. 2021; Yao et al. 2021): *C.guangxiensis* (Yin & Tan, 2000) (Guangxi, Hainan), *C.hubei* Song & Zhao, 1988 (Hubei, Chongqing), *C.liboensis* Zhu & Zhang, 2008 (Guizhou, Guangxi) and *C.lubricus* Yu et al., 2021 (Yunnan). Here, we describe two new species: *Chilobrachysdominus* sp. nov. from Xishuangbanna, Yunnan and *Chilobrachysjinchengi* sp. nov. from Medog, Tibet.

## ﻿Materials and methods

All specimens are preserved in 75% ethanol and deposited in the Institute of Zoology, Chinese Academy of Sciences in Beijing (**IZCAS**). Spermathecae were cleared in a trypsin enzyme solution to dissolve non-chitinous tissues. Specimens were examined under a LEICA M205C stereomicroscope. Photomicroscope images were taken with an Olympus C7070 zoom digital camera (7.1 megapixels). Photographs were stacked with Helicon Focus 6.7.1 and processed in Adobe Photoshop CC 2018.

The terminology used in the text and figures follows Bertani (2000). All measurements are in millimetres. Eye sizes were measured as the maximum diameter in either a dorsal or frontal view. Leg measurements are given as follows: total length (femur, patella, tibia, metatarsus, tarsus).

### ﻿Abbreviations

**A** apical keel;

**ALE** anterior lateral eyes;

**AME** anterior median eyes;

**MOA** median ocular area;

**PI** prolateral inferior keel;

**PLE** posterior lateral eyes;

**PME** posterior median eyes;

**PS** prolateral superior keel.

## ﻿Taxonomy

**Family Theraphosidae Thorell, 1869**

### ﻿Subfamily Selenocosmiinae Simon, 1889

#### ﻿Genus Chilobrachys

Taxon classificationAnimaliaAraneaeTheraphosidae

Karsh, 1892

2C5BA966-A125-5ED0-A086-259D174C0332

##### Type species.

*Chilobrachysnitelinus* Karsch, 1892.

##### Diagnosis.

See Raven (1985) and Zhu and Zhang (2008).

#### ﻿ Chilobrachys dominus

Taxon classificationAnimaliaAraneaeTheraphosidae

Lin & Li sp. nov.

554A502C-BC8A-580F-9EE1-1CDE32F5A2FF

http://zoobank.org/36EC608E-6671-407E-9E1D-10C5497BADC0

**Figure 1.**
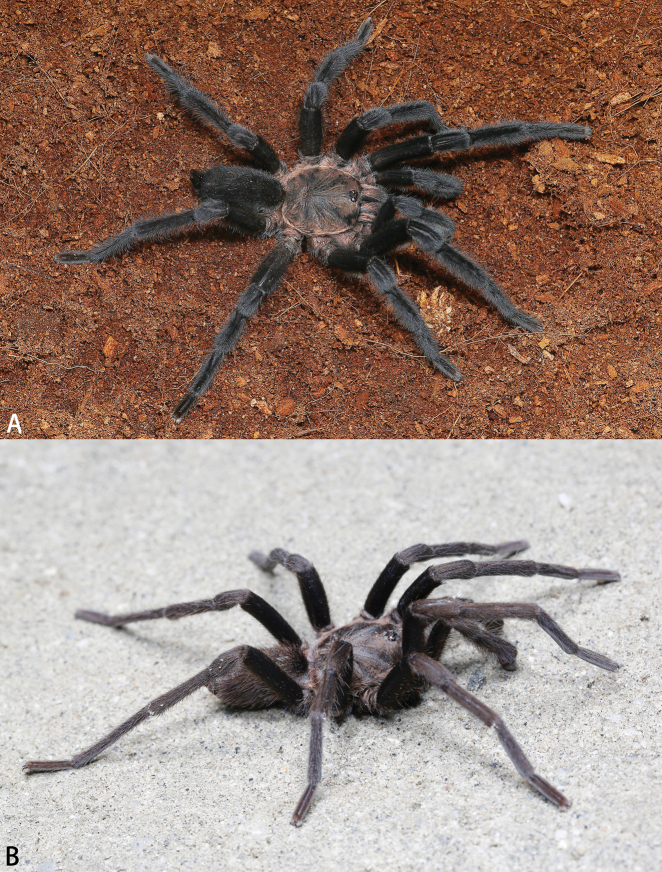
*Chilobrachys* spp. nov., live males **A***Chilobrachysdominus* sp. nov. **B***C.jinchengi* sp. nov. Photos by Qianle Lu, Zhengzhong Huang.

**Figure 2.**
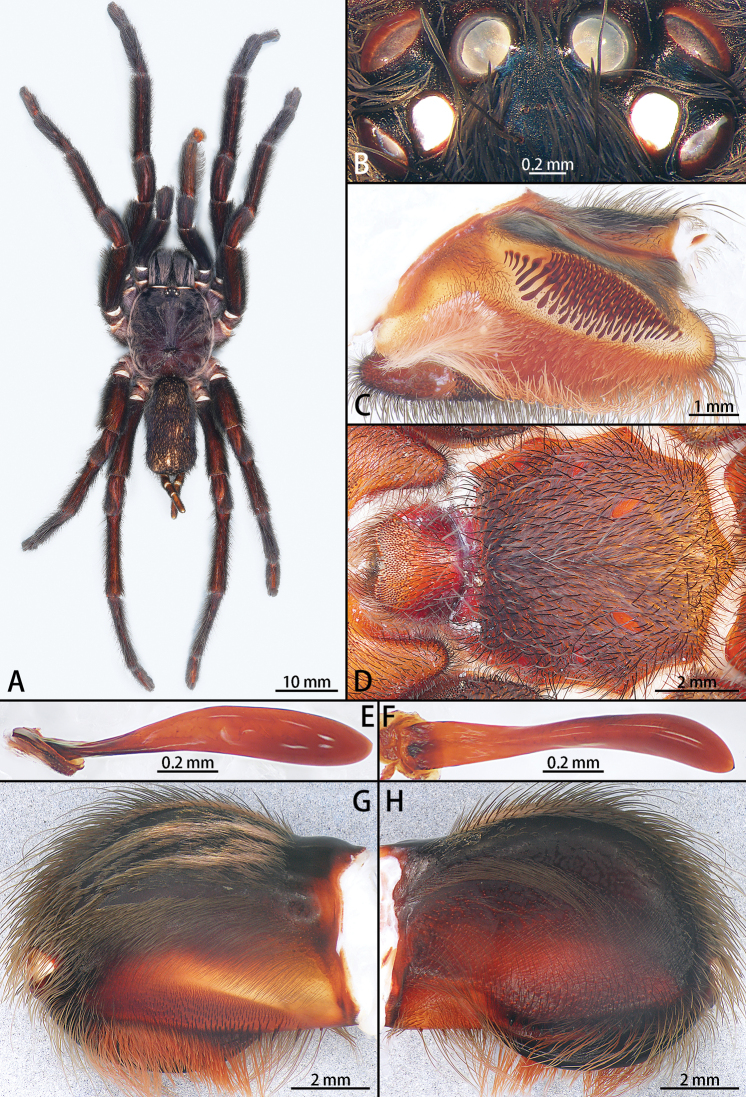
*Chilobrachysdominus* sp. nov., holotype male **A** male habitus, dorsal **B** ocular tubercle **C** right palp maxillae **D** sternum **E** stridulatory lyra, lateral view **F** same, ventral view **G** chelicerae, retrolateral view **H** same, prolateral view.

**Figure 3.**
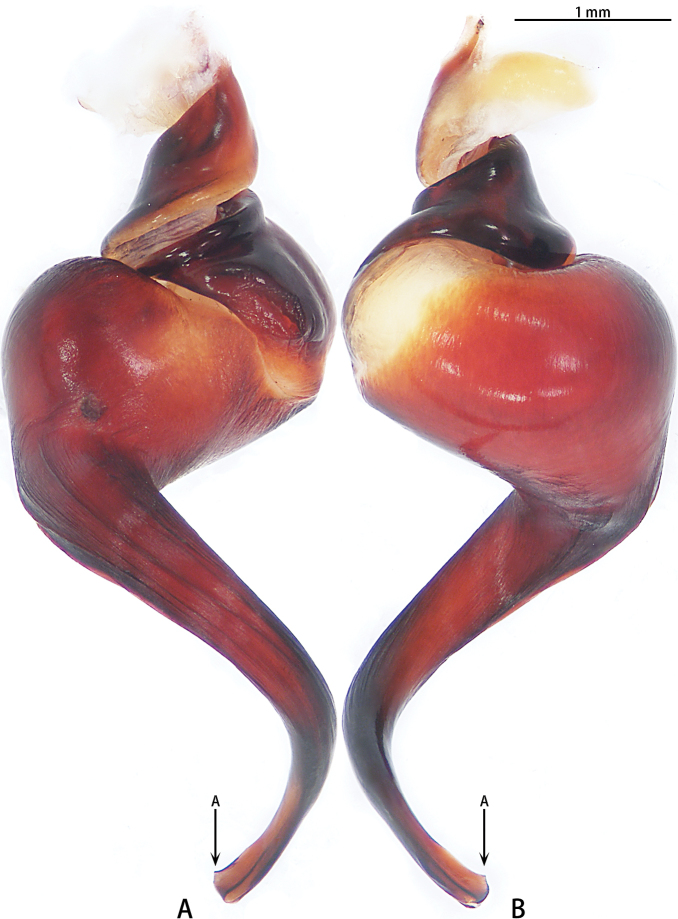
*Chilobrachysdominus* sp. nov., holotype, right palp bulb, rotated horizontally **A** retrolateral view **B** prolateral view.

**Figure 4.**
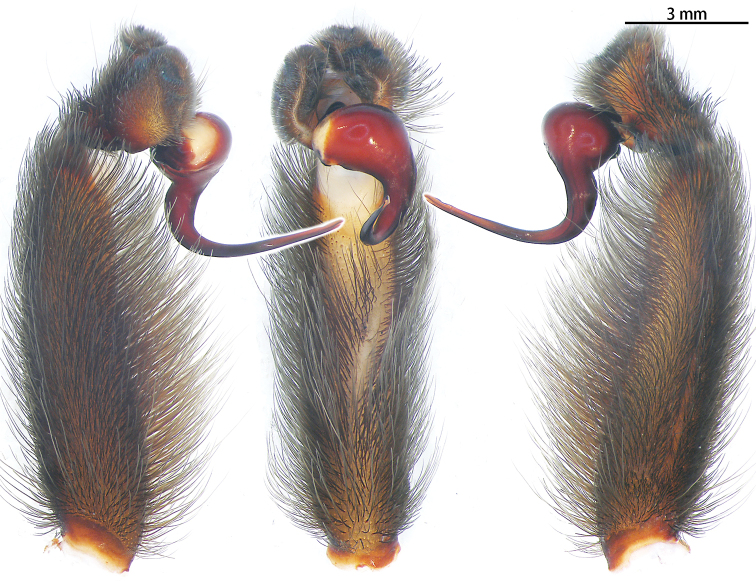
*Chilobrachysdominus* sp. nov., holotype, left palp **A** prolateral view **B** ventral view **C** retrolateral view.

Figs 1A , 2A–H , 3A, B , 4A–C , 8

##### Type material.

***Holotype*** ♂ (IZCAS-Ar42676), **China: *Yunnan***: Jinghong, Mount Jinuo, Jinuo Road, 22.0556°N, 100.9853°E, elevation 1040 m, 16.XI.2021, Yi Ming leg.

##### Diagnosis.

The male of *Chilobrachysdominus* sp. nov. is similar to that of *C.pococki* (Thorell, 1897) in having a long, strongly curved embolus, but can be distinguished by the embolus bent in at two thirds (vs. halfway in *C.pococki*).

##### Description.

**Male** (holotype, IZCAS-Ar42676) (Figs 2B–H, 3, 4). Carapace 13.27 long, 11.89 wide, black, with long purple setae. Opisthosoma black, with long black setae. Eye group 2.08 long, 1.04 wide (Fig. 2F). MOA 0.81 long, anterior width 1.05, posterior width 1.53. Eye sizes and interdistances: ALE 0.52, AME 0.44, PLE 0.37, PME 0.37; ALE–AME 0.17, AME–AME 0.24, PLE–PME 0.02, PME–PME 0.90. Fovea slightly procurved. Chelicerae black, with row of 14 promarginal teeth. Labium wider than long, with 368 cuspules. Sternum yellow brown with 3 pairs of sigilla. Legs with long and short setae. Tarsi I–III with 2 claws, tarsus IV with 3 claws, denticle number: I 4, II 6, III 5, IV 4. Leg measurements: I 43.77 (12.70 + 5.62 + 11.52 + 7.39 + 6.54), II 38.16 (10.54 + 4.88 + 9.92 + 7.63 + 5.19), III 35.95 (9.59 + 4.08 + 7.87 + 8.61 + 5.80), IV 48.11 (12.72 + 4.89 + 11.10 + 12.75 + 6.65). Leg formula: 4123.

Male palpal bulb (Fig. 3A, B; male palp with bulb Fig. 4A–C). Maxillae with lyra setae ventrally. Bulb oval, embolus slender and long, strongly curved at 90°, with A.

**Female.** unknown.

##### Etymology.

The species is named after the Latin word *dominus*, as this is one of the rarest spiders from China; noun (name) in apposition.

##### Distribution.

Known only from the type locality.

#### ﻿ Chilobrachys jinchengi

Taxon classificationAnimaliaAraneaeTheraphosidae

Lin & Li sp. nov.

FC2BA48D-1D5A-5726-9A67-44DCFCA49589

http://zoobank.org/CA76A9EC-61AF-4EBA-92F5-521EF797E229

**Figure 5.**
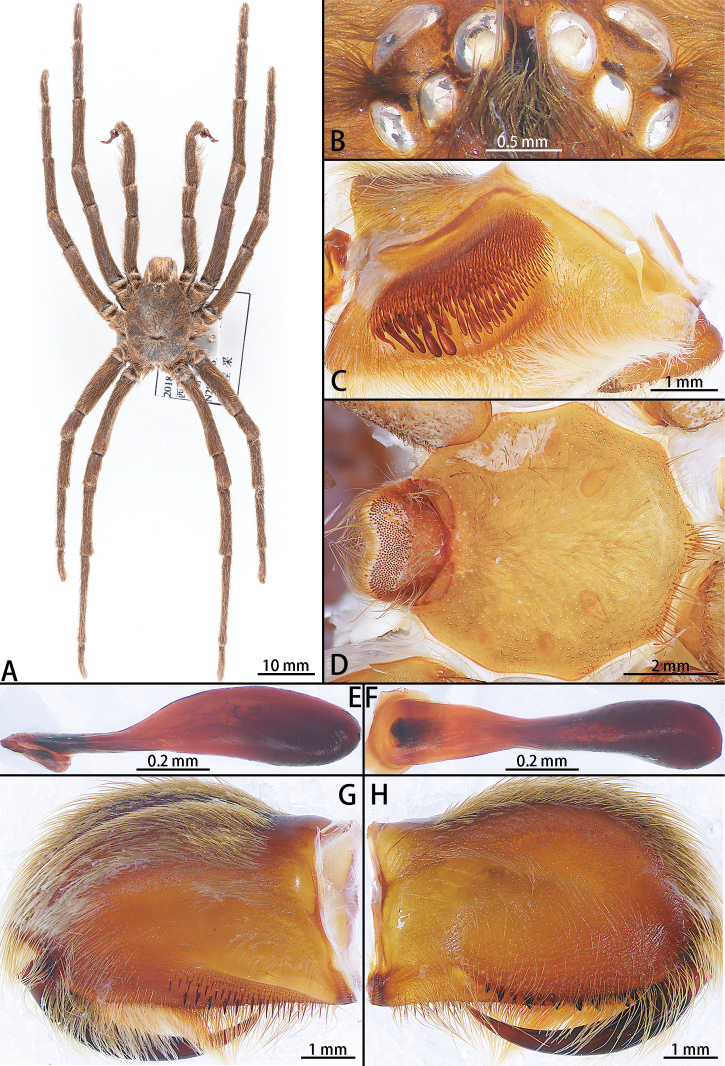
*Chilobrachysjinchengi* sp. nov., holotype (**B–H**) and paratype (**A**) males **A** male habitus, dorsal **B** ocular tubercle **C** right palp maxillae **D** sternum **E** stridulatory lyra, lateral view **F** same, ventral view **G** chelicerae, retrolateral view **H** same, prolateral view.

**Figure 6.**
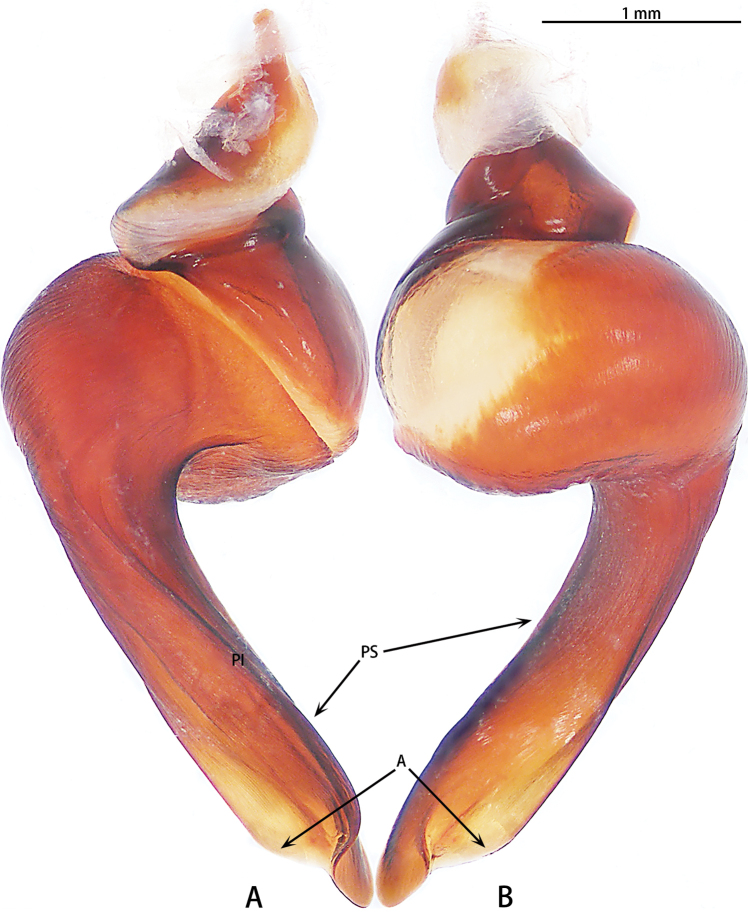
*Chilobrachysjinchengi* sp. nov., holotype, right palp bulb, rotated horizontally **A** retrolateral view **B** prolateral view.

**Figure 7.**
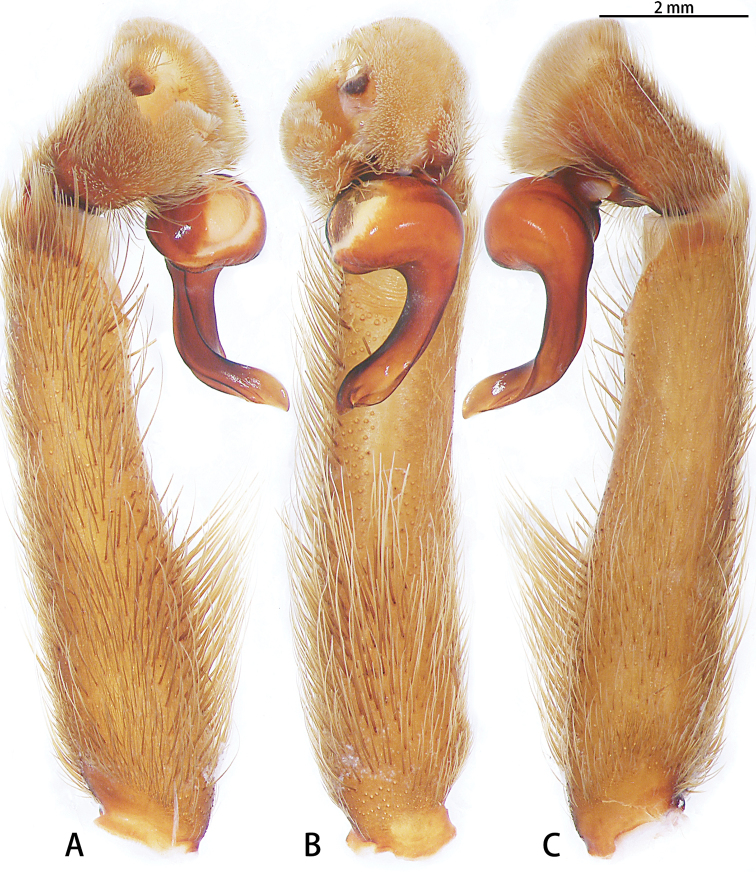
*Chilobrachysjinchengi* sp. nov., holotype, left palp **A** prolateral view **B** ventral view **C** retrolateral view.

**Figure 8.**
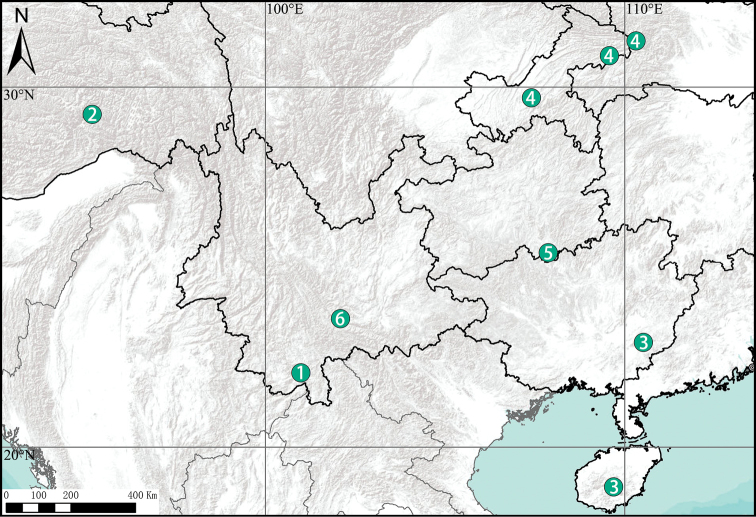
Distribution records of *Chilobrachys* species in China **1***C.dominus* sp. nov. **2***C.jinchengi* sp. nov. **3***C.guangxiensis* (Yin & Tan, 2000) **4***C.hubei* Song & Zhao, 1988 **5***C.liboensis* Zhu & Zhang, 2008 **6***C.lubricus* Yu et al., 2021.

Figs 1B , 5A–H , 6A, B , 7A–C , 8

##### Type material.

***Holotype*** ♂ (IZCAS-Ar42677), **China: *Tibet***: Nyingchi, Medog, from Baibung to Muge, 29.2386°N, 95.1831°E, elevation 1310 m, 21.VIII.2018, Jincheng Liu leg. ***Paratype*** 1♂ (IZCAS-Ar42678), same data as holotype.

##### Diagnosis.

The male of *Chilobrachysjinchengi* sp. nov. is similar to that *C.bicolor* (Pocock 1895), but can be distinguished by the length ratio of the embolus to the bulb, which is almost 2:1 in *C.jinchengi* sp. nov. and is 1:1 in *C.bicolor*.

##### Description.

**Male** (holotype, IZCAS-Ar42677) (Figs 5B–H, 6, 7). Carapace 13.12 long, 10.21 wide, brown with long setae. Opisthosoma absent. Eye group 2.32 long, 0.97 wide (Fig. 5F). MOA 0.77 long, anterior width 0.89, posterior width 1.38. Eye sizes and interdistances: ALE 0.51, AME 0.45, PLE 0.42, PME 0.38; ALE–AME 0.34, AME–AME 0.25, PLE–PME 0.12, PME–PME 0.87. Fovea slightly procurved. Chelicerae dark brown, with row of 12 promarginal teeth. Labium wider than long, with 367 cuspules. Sternum yellow-brown, with 3 pairs of sigilla. Legs with long and short setae. Tarsi I–III with 2 claws, tarsus IV with 3 claws, denticle number: I 9, II 9, III 9, IV 12. Leg measurements: I 49.58 (14.23 + 7.12 + 11.21 + 9.89 + 7.13), II 45.77 (13.02 + 6.73 + 9.52 + 9.59 + 6.91), III 43.21 (11.51 + 5.48 + 8.24 + 11.51 + 6.47), IV 53.97 (14.32 + 5.87 + 11.22 + 15.96 + 6.60). Leg formula: 4123.

Male palpal bulb (Fig. 6A, B; male palp with bulb Fig. 7A–C). Maxillae with lyra setae ventrally. Bulb oval, embolus bow-shaped, strongly curved at 180°, with A, PI and PS. Distal edge of embolus relatively flat.

**Female.** unknown.

##### Etymology.

The species is named after Mr. Jincheng Liu, who collected the type material; noun (name) in the genitive case.

##### Distribution.

Known only from the type locality.

## Supplementary Material

XML Treatment for Chilobrachys

XML Treatment for Chilobrachys dominus

XML Treatment for Chilobrachys jinchengi

## References

[B1] Bertani R (2000) Male palpal bulbs and homologous features in Theraphosinae (Araneae, Theraphosidae). Journal of Arachnology 28: 29–42. 10.1636/0161-8202(2000)028[0029:MPBAHF]2.0.CO;2

[B2] Foley S Lüddecke T Chen DQ Krehenwinkel H Künzel S Longhorn S Wendt I von Wirth V Tänzler R Vences M Piel WH (2019) Tarantula phylogenomics: A robust phylogeny of deep theraphosid clades inferred from transcriptome data sheds light on the prickly issue of urticating setae evolution. Molecular Phylogenetics and Evolution 140: 106573. 10.1016/j.ympev.2019.106573 31374259

[B3] Li J Yan X Lin Y Li S Chen H (2021) Challenging Wallacean and Linnean shortfalls: *Ectatosticta* spiders (Araneae, Hypochilidae) from China. Zoological Research 42(6): 791–794. 10.24272/j.issn.2095-8137.2021.212 PMC8645878 34704425

[B4] Li S (2020) Spider taxonomy for an advanced China. Zoological Systematics 45(2): 73–77. 10.11865/zs.202011

[B5] Nunn SC West RC von Wirth V (2016) A revision of the selenocosmiine tarantula genus *Phlogiellus* Pocock 1897 (Araneae: Theraphosidae), with description of 4 new species. International Journal of Zoology 2016: e9895234. [54 pp.] 10.1155/2016/9895234

[B6] Pocock RI (1895) On a new and natural grouping of some of the Oriental genera of Mygalomorphae, with descriptions of new genera and species. Annals and Magazine of Natural History (6) 15(86): 165–184. 10.1080/00222939508677863

[B7] Raven RJ (1985) The spider infraorder Mygalomorphae (Araneae): cladistics and systematics. Bulletin of the American Museum of Natural History 182: 1–180.

[B8] Simon E (1889) Arachnides. In Voyage de M. E. Simon au Venezuela (décembre 1887–avril 1888). 4^e^ Mémoire. Annales Société entomologique de France, Paris (6)9: 169–220.

[B9] WSC (2021) World Spider Catalog. Version 22.5. Natural History Museum Bern. http://wsc.nmbe.ch [accessed on 21/10/2021] 10.24436/2

[B10] Yao Z Wang X Li S (2021) Tip of the iceberg: species diversity of *Pholcus* spiders (Araneae, Pholcidae) in Changbai Mountains, Northeast China. Zoological Research 42(3): 267–271. 10.24272/j.issn.2095-8137.2020.214 33797209 PMC8175958

[B11] Zhu MS Zhang R (2008) Revision of the theraphosid spiders from China (Araneae: Mygalomorphae). Journal of Arachnology 36: 425–447. 10.1636/CA07-94.1

